# Predicting Individual Function During COVID-19 Lockdown: Depression, Fear of COVID-19, Age, and Employment

**DOI:** 10.3389/fpsyg.2021.682122

**Published:** 2021-07-01

**Authors:** Inna Levy, Keren Cohen-Louck

**Affiliations:** ^1^Department of Criminology, Ariel University, Ariel, Israel; ^2^Department of Interdisciplinary Studies, Zefat Academic College, Safed, Israel

**Keywords:** age, COVID-19, depression, employment, fear of COVID-19, function, unemployment

## Abstract

This study aims to identify the significance of age and employment to individual function during COVID-19. An online survey included 509 Israeli citizens, ages 18–78, who reported individual function, depression, fears related to COVID-19 and demographic characteristics. Structural Equation Modeling (SEM) analysis showed a good fit between our model and the data. Age and employment were negatively associated with depression and economic fears related to COVID-19 that, in turn, were negatively associated with individual function. The effect of age and employment on individual function was fully mediated *via* depression and economic fears related to COVID-19. The discussion addresses our findings in the context of the victimization paradox. Although COVID-19 related health complications are more frequent among older adults, our results suggest that practitioners responsible for public mental health during viral pandemics should consider young age and unemployment as risk factors for depression and low individual function.

## Introduction

The COVID-19 pandemic started in December 2019 in Wuhan, China (Ren et al., 2020) and caused a wave of viral victimization or *viruism* (Cohen-Louck and Levy, 2020). Viruism is an array of pandemics' adverse effects on individual physical and mental health, economy, and civil rights. It is essential to extend the knowledge on the social and psychological aspects of viruism to improve coping with future pandemics and the related quarantines (for review of viruism, see Cohen-Louck and Levy, 2020). Thus, one of this study's goals was to assess Israeli citizens' levels of individual function as well as depression and fear of COVID-19 during coronavirus lockdown.

Individual functioning represents the individual quality of life (Suurmeijer et al., 2002) and includes psychological aspects of functioning and job, family, and social performance (Altshuler et al., 2002). Considering the clinical significance of individual functioning (e.g., Hui et al., 2013; Tang and Thomas, 2020), the central aim of this study is to examine the model that predicts individual functioning during the virus-related lockdown and identify the inter-relationships between depression, fears of COVID-19, age and employment.

### Depression, Individual Functioning, and Fear

COVID-19 related illness, deaths, and quarantine measures affect many aspects of people's lives and cause depression, anxiety, and stress (Mariani et al., 2020; Qiu et al., 2020; Shapiro et al., 2020; Wang et al., 2020; Braun-Lewensohn et al., 2021). Although some studies claimed that they examined individual function, they did not address function, *per se*, but defined function through such concepts as stress, mental health, and resilience (e.g., Mimoun et al., 2020; Kavčič et al., 2021; Kocjan et al., 2021). Thus, the research on individual function during pandemics is limited, and the current study intends to fill this gap by addressing the association between function, depression, fears of COVID-19, and age, and employment.

Prior research shows that depression has adverse effects on the overall quality of life (Wada et al., 2005; Tang and Thomas, 2020) and individual function (Jaycox et al., 2009). The effects of depression on function are long, lead to interpersonal and social difficulties (Schottenbauer et al., 2008; Dorahy et al., 2017) and unsatisfactory family and marital functioning (Bromberger and Lanza di Scalea, 2009; Restifo and Bögels, 2009). Moreover, treatment of depressive symptoms is associated with functional recovery (Oluboka et al., 2018). Thus, it is possible to assume that depression is associated with diminished function.

Additionally to depression, the COVID-19 pandemic caused exceptionally high levels of fear (Ho et al., 2020) due to its simultaneous worldwide spread, high media attention, lack of public knowledge and effective medical treatment, and drastic and unprecedented preventive measures (e.g., lockdowns and quarantine; Ren et al., 2020). Therefore, the pandemic affected not just sick but also healthy citizens who felt unsafe, uneasy, and anxious (Cohen-Louck and Levy, 2020; Shigemura et al., 2020). The fears related to the COVID-19 pandemic include not just fears of contracting the virus and fear about family members' health (Ahorsu et al., 2020; Liu et al., 2020; Presti et al., 2020; Shapiro et al., 2020) or fears of death (Horesh and Brown, 2020). The COVID-19-related fears also refer to the possible adverse economic outcomes due to the lockdowns (Presti et al., 2020; Shapiro et al., 2020). All these fears cause, inter alia, emotional distress, depression, and diminished life satisfaction (Li et al., 2020; Presti et al., 2020). Furthermore, there is a negative association between fears and psychosocial function (Pat-Horenczyk et al., 2007; Stafford et al., 2007). For example, fear of crime and fear of terrorism are associated with cognitive and functional impairment in social, family, and health-related domains, including decreased contact with friends and reduced involvement in social and physical activities (Tanasescu, 2002; Warburton, 2006; Pat-Horenczyk et al., 2007; Stafford et al., 2007). Therefore, we assume that fears of COVID-19 will be negatively associated with individual function.

### Individual Functioning, Age, and Employment

The ramifications of the COVID-19 pandemic emphasized the significance of age and employment. COVID-19 caused high mortality rates among older adults and raised a concern that they might also be at high risk of psychological distress and depression (Qiu et al., 2020). In fact, some studies show that the levels of depression (Wilchek-Aviad and Cohen-Louck, 2020) and fear (Shechory-Bitton and Cohen-Louck, 2020) are higher among older adults than young adults. Nevertheless, many studies indicate a decline in anxiety and fear with aging and argue that aging is a protective factor (Russac et al., 2007; Charles and Carstensen, 2010; Mather, 2016). COVID-19 studies also reinforce this notion and show that younger individuals experience greater depression and anxiety than older adults (Gualano et al., 2020; Moccia et al., 2020; Braun-Lewensohn et al., 2021; Shechory Bitton and Laufer, 2021). Based on these findings, we suggest that age will be negatively associated with depression and fears and positively associated with function.

Additionally to age, this study addressed employment because, during COVID-19 lockdowns, many people lost their jobs (Estrada and Arturo, 2020). Generally, unemployed are at higher risk for depression and tend to report lower satisfaction within life domains (Lau et al., 2008; Chiao et al., 2011; Melchior et al., 2013). Furthermore, during the COVID-19 pandemic, unemployed people exhibited high levels of anxiety and depression, diminished well-being (Holmes et al., 2020; Kuang et al., 2020), and poor function (e.g., Kazmi et al., 2020; Moccia et al., 2020). Thus, it seems that unemployment is related to emotional distress (e.g., depression and fears) and decreased individual function.

### Current Research Aim and Hypotheses

**Figure 1:**
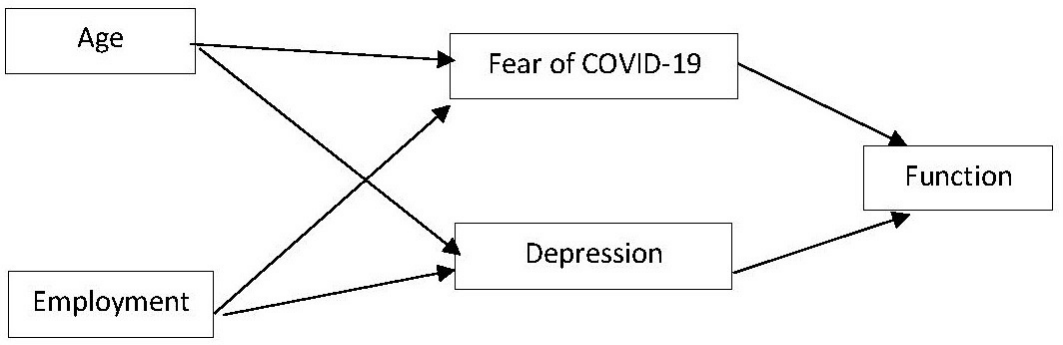
Theoretical model for predicting function during COVID-19 quarantine.

The current research aims to identify factors predicting individual functioning during the pandemic-related lockdown. This study focuses on a model (Figure 1) that investigates the relationship between depression, fear of COVID-19, age, employment, and individual function. The literature review indicates that age and employment are associated with depression and fears, while depression and fears are associated with individual function. Therefore, based on the literature review, the hypotheses were:

H_1_: *Depression is negatively associated with individual function among people who experience pandemic-related lockdown*.
H_2_: *COVID-19 related fears are positively associated with depression*.
H_3_: *COVID-19 related fears are negatively associated with individual function*.
H_4_: *Age is negatively associated with depression and fear of COVID-19 and positively associated with function*.
H_5_*: Employment is negatively associated with depression and fear of COVID-19 and positively associated with function*.
H_6_: *Depression and fear of COVID-19 mediate the relationship between age and function*.
H_7_: *Depression and fear of COVID-19 mediate the relationship between employment and function*.

## Methods

### Participants

This study included 509 Israeli respondents. Respondents' age range was 18–78 [Mean = 41.61, S.D. = 15.55]. Table 1 presents a detailed demographic description of the sample. About half of the respondents were male and reported low than average income for a household. More than half reported that there are children in the household. As for education, more than half (57.2%) reported an academic level of education. The majority were Jewish, married and defined themselves as secular.

**Table 1:** Participants demographic characteristics (*N* = 509).

**Demographic characteristics**	**%**
Gender	
Male	50.7
Female	49.3
Family status	
Single	23.8
Living with partner	7.70
Married	60.10
Divorced	7.10
Widows	1.40
Children (under age of 18) at the household	
Yes	53.8
No	46.2
Number of children at the household	
1 child	32.1
2 children	28.1
3 children	22.3
4 children	8.0
5+ children	9.5
Educational level	
Primary-high school	22.8
Tertiary level	19.8
B.A. (or equivalent) level	38.7
M.A.	16.9
Ph.D.	1.6
Nationality* religion	
Arab	12
Druze	3.9
Jewish	84.1
Religiosity	
Secular	47.9
Traditional	32.0
Religious	13.1
Orthodox	7.0
Household income in NIS	
3,000–9,000	24.2
9,001–14,000	25.4
14,001–22,000	28.0
22,001–36,000+	22.4
Employment during Covid-19	
Working as usual	26.7
Working from home	24.6
Leave without pay	22.0
Fired	5.9
Did not work before Covid-19	10.0
Retired	10.8

Table 2 compares our sample and the Israeli population by gender, ethnicity, and religiosity. Table 2 indicates that the sample matches the Israeli population by gender and religiosity.

**Table 2:** Distribution of gender, ethnicity, religiosity, and educational levels in this study's sample (*N* = 509) and in the Israeli population.

	**Current sample**	**Israeli population**	**χ^2^**	***df***	***p***	***Cramer's V***
Gender^a^^,^^c^						
Male	50.7%	49.5%	0.29	1	0.59	0.002
Female	49.3%	50.5%				
Ethnicity^a^^,^^d^						
Arabs (including Druze)	15.9%	22%	2	1	0.00	0.15
Jews	84.1%	78%				
Religiosity^b^^,^^e^						
Secular	47.9%	43.2%	4.27	1	0.04	0.09
Religious	52.1%	56.4%				

a *The data was calculated based on the publication of the Israeli Central Bureau of Statistics (N = 8,967.6 thousands; Central Bureau of Statistics, 2019)*.

b *Central Bureau of Statistics (2020)*.

c *The difference between the distribution of gender in the sample and in the population is non-significant*.

d *Although in general the distribution of ethnicity in our sample resembles the distribution in the population, due to the big sample size the difference between the distribution of ethnicity in the sample and in the population is significant, and the magnitude of the difference is medium*.

e *The difference between the distribution of religiosity in the sample and in the population is significant, but the magnitude of the difference is small*.

### Measures

#### Demographic Variables

The questionnaire gathered information on participants' gender, age, family status, number of children in a household, educational level, religiosity, religion, income, and employment (for variables values, see Table 1).

#### Function

Psychotherapy Outcome Assessment and Monitoring System–Trauma Version (POAMS-TV) questionnaire (Green et al., 2003) includes 10 items on functioning in different life spheres: work, study, partner relationships, relationships with children, social activities, friendships, sexual functioning, quality of life, health and financial management. Each item is rated on a 5-point scale ranging from 0 (extreme distress or dissatisfaction) to 4 (optimal functioning or satisfaction). A score of three or more suggests healthy functioning. A global functioning score was obtained by averaging across items. The item on relationships with children was excluded because it was relevant only to parents. Cronbach's alpha was 0.85.

#### Depression

The depression subscale from the short Depression, Anxiety and Stress Scale (DASS-21; Lovibond and Lovibond, 1995) was used. Respondents scored seven items on a scale from 0 (did not apply to me at all) to 3 (apply to me very much). Sum scores are computed by adding up the items and multiplying them by a factor of 2. Thus, the sum scores for the depression subscale may range between 0 and 42. According to Lovibond and Lovibond (1995), scores from 0 to 9 are considered normal, 10–13 mild depression, 14–20 moderate, 21–27 severe, and 28+ extremely severe. Cronbach's alpha was 0.89.

#### Fear of COVID-19

**Figure 2:**
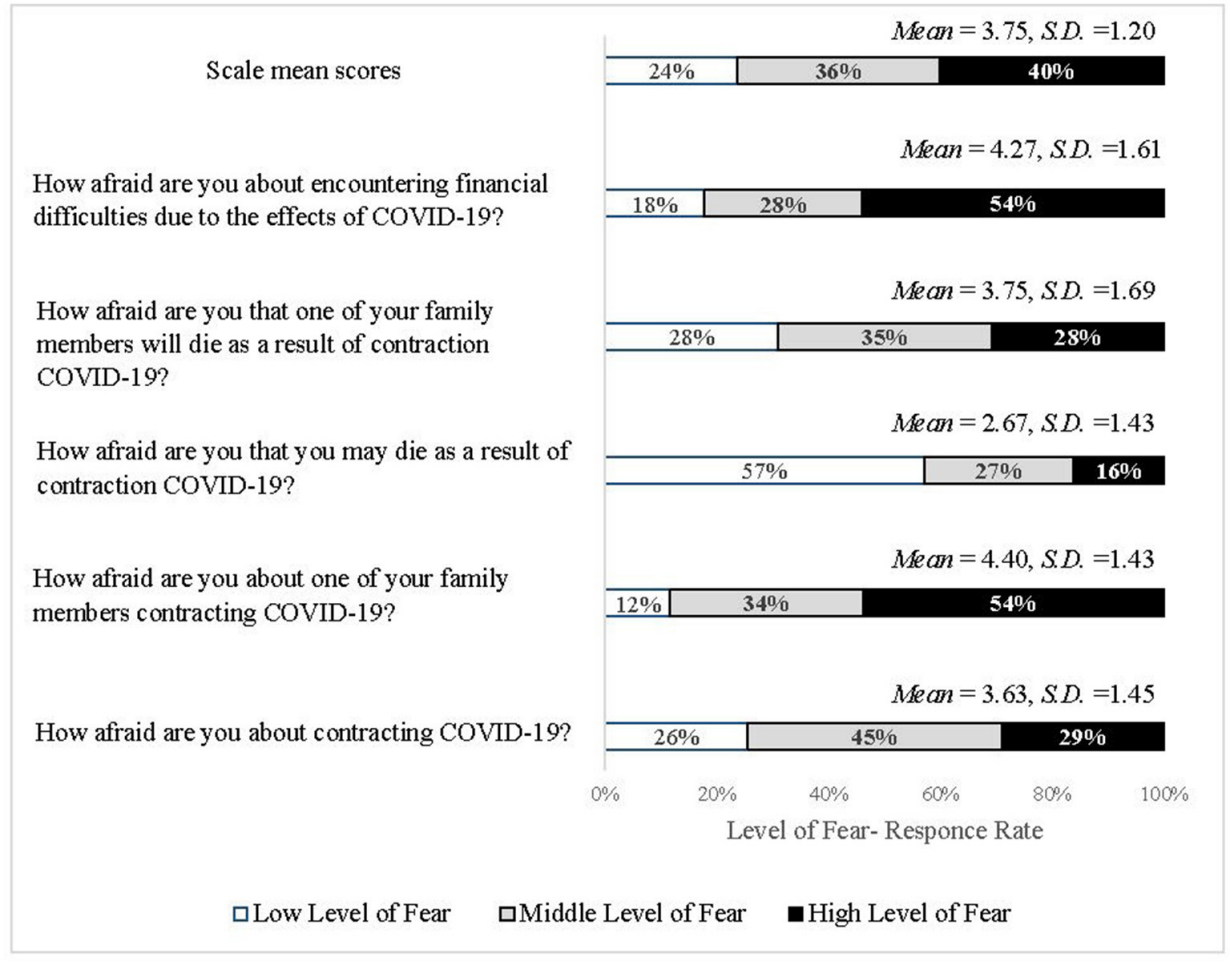
Distribution of responses, means and standard deviations on fear of COVID-19 items (*N* = 509).

In the absence of existing scales at the time of the survey, to examine the fear of COVID-19, the authors constructed a five items scale on fear of COVID-19 health-related and economic effects (Figure 2). The items in this scale are based on the items from studies on fear of crime and fear of terrorism (Klar et al., 2002; Franklin et al., 2008). The participants scored each item on a scale of 1 (not afraid at all) to 6 (extremely afraid). Cronbach's alpha was 0.83.

### Procedure

#### COVID-19 at the Time of Data Collection

In Israel, the first case of COVID-19 was identified on February 21, 2020. Starting from March 10, 2020, the Israeli government gradually limited gatherings and imposed increasing restrictions. By March 13, all educational institutions, day centers, after school programs were closed (Stein-Zamir et al., 2020). On March 19, due to the daily growing number of confirmed cases, the government ordered the closure of all non-essential businesses, increased social distancing, and significantly limited citizens' movements outside of their homes (Last, 2020). The first lockdown lasted from March 16 to April 19, 2020 (Birenbaum-Carmeli and Chassida, 2020).

#### Data Collection

The data represent respondents' self-reports through an online survey during the first lockdown, from 22 March 2020 to 26 March 2020. The University Ethical committee provided ethical approval. The questionnaire stated that participation was anonymous and confidential, that the participants did not have to answer any question that made them uncomfortable and could stop answering at any point, and that their answers would serve only for research purposes. All participants gave their informed consent to participate in this study. The sampling from the panel was random. The sample matches the Israeli population in the distribution of such sociodemographic characteristics as gender, age, nationality (Arabs/Jews), religiosity, residential area, and Arabs' religion (Christian, Druze, Muslim). A survey company collected the data through an online panel. To ensure the survey's correct visual presentation, the participants could answer the survey only *via* personal computers. The participants were paid 100 NIS (about 30$) for their participation because the survey included many questions.

The sampling was based on a matrix created by a combination of such variables as living area (area code), gender, age, and religiosity level. The different combinations of these variables' values create small groups of compound characteristics. For example, there is a group of participants who are “male, age 18–21, secular, from 04 area code (North Israel)” and a group of participants who are “male, age 18–21, secular, from 03 area code (Central Israel).” For each group, there is a specific quota of respondents. When the quota is reached, there is no further sampling of respondents with such characteristics. This methodology allows creating samples that match the characteristics of Israeli populations. The panel includes 130,000 potential respondents. To sample about 500 participants, the survey company sent 5,000 invitations to the individuals listed on this panel. In online studies, those respondents who answer quickly are included in the study. Thus, out of 5,000 invitations, only the first 509 responders had a chance to participate. Those who did not participate cannot be compared to potential participants who refused to answer a phone/door-to-door survey. They may have been slower and might have responded later if the slots were not filled. Thus, the issue of non-response bias is less relevant to this type of sampling. Furthermore, research shows only modest differences in outcomes between samples with high and low response rates (Curtin et al., 2000; Fosnacht et al., 2017). The decision to sample about 500 respondents addressed that the Israeli population is close to 9 million, with a 95% confidence level and a 4.4% confidence interval.

#### Data Analysis

The analyses were carried out using SPSS Version 25 and AMOS24. This study used *t*-tests, univariate and repeated-measures ANOVA, Pearson correlation and Chi-square analysis to assess the differences in functioning, depression, and fear of COVID-19 by demographic characteristics. To conduct the analysis, we used the continuous scores on the depression scale, but we also assessed the discrete results to evaluate the levels of depression within the population. The employment was re-coded into a demi variable: 0 = not working, 1 = working. Model fit was estimated using Structural Equation Modeling (SEM). The analysis addressed the following indices: CFI, TLI, RMSEA, SRMR (McCoach et al., 2013), and CMIN/DF ratio (Hinz et al., 2017). Cutoff values of >0.95 indicate a good fit for CFI and TLI, with values <0.06 for RMSEA and SRMR (Hu and Bentler, 1999). The ratio for χ^2^/df should be <3 (Akbar and Parvez, 2009). The authors employed PROCESS v.3.4 (model 4) for mediation, a widely preferred tool for testing indirect or mediation effects (MacKinnon et al., 2007).

## Results

### Descriptive Findings

The sample mean of function was 2.24 [*S.D*. = 0.84, *Range* = 0–4] indicating a relatively low level of function. The mean score for depression was 9.34 [*S.D*. = 9.43, *Range* = 0–42]: 59.5% of respondent reported normal levels (Lovibond and Lovibond, 1995), 12.6% mild levels, 16% moderate levels, 6% severe, and 5.9% extremely severe levels of depression. As for COVID-19 related fears (Figure 2), repeated measures ANOVA indicated that there was a significant difference between types of fears [*F*_(4, 505)_ = 177.19, η^2^ = 0.58, *p* < 0.001]. The strongest fear was that a family member would be infected with COVID-19, followed by the fear of economic harm. In the third place was the fear that a family member will die due to the virus; in the fourth place was the fear of getting infected, and the weakest fear was to die because of the virus.

Table 3 presents the association between demographic variables and fear of COVID-19, depression and function. There were significant differences in fear of COVID-19 by the presence of children at the household, religiosity and educational level. Respondents who have children living with them, secular and traditional and with the academic level of education, reported higher levels of fears of COVID-19 than those who have children in their households, are religious and do not have an academic education. There were no significant differences in fear of COVID-19 by gender, family status and nationality. In depression, the only significant difference was by family status. The two significant pair-wise comparisons were between married and single respondents and those who live with a partner. Married respondents reported significantly lower levels of depression than respondents who were single or lived with a partner. There were no significant differences in depression by gender, living with children, nationality, religiosity, and educational level. As for function, the only significant difference was by family status and religiosity. The *post-hoc* analyses (Sheffe) indicated that the only significant pair-wise comparison was between married and single respondents. Married respondents reported the highest level of function, and single respondents reported the lowest level of function. As for religiosity, secular reported the lowest function level, traditional—middle level, and religious reported the highest of function. The difference was significant only between secular and religious respondents. There were no significant differences in function by gender, children in the household, nationality and educational level.

**Table 3:** Association between demographic characteristics, function (POAMS-TV) and depression (*N* = 509).

	**Fear of COVID-19**	**Depression**	**Function**
	**Mean (S.D.)**	**Mean (S.D.)**	**Mean (S.D.)**
In the sample	3.75 (1.20)	8.90 (9.44)	2.44 (0.85)
Gender			
Female (*n* = 251)	3.80 (1.21)	9.90 (9.27)	2.44 (0.86)
Male (*n* = 258)	3.69 (1.18)	8.78 (9.58)	2.43 (0.83)
*t*	−1.196	−1.34	−0.11
*df*	507	507	507
Family status			
Single (*n* = 121)	3.86 (1.24)	12.17 (10.10)	2.21 (0.76)
Living with a partner (*n* = 39)	4.07 (1.11)	14.05 (11.11)	2.16 (0.91)
Married (*n* = 306)	3.69 (1.21)	8.51 (8.51)	2.56 (0.85)
Divorced (*n* = 36)	3.49 (1.57)	8.77 (9.29)	2.44 (0.78)
Widows (*n* = 7)	3.66 (1.57)	5.43 (9.14)	2.49 (0.97)
*F*	1.58	8.01^***^	4.77^**^
*df*	4, 504	4, 504	4, 504
Children at the household			
Yes (*n* = 274)	3.85 (1.89)	8.73 (8.73)	2.43 (0.79)
No (*n* = 235)	3.62 (1.21)	10.19 (10.15)	2.44 (0.90)
*t*	2.25^*^	−1.89	−0.08
*df*	507	507	507
Nationality			
Arabs (*n* = 81)	3.84 (1.13)	11.06 (10.37)	2.30 (0.82)
Jews (*n* = 428)	3.73 (1.51)	9.01 (9.23)	2.47 (0.85)
*t*	0.85	1.70	−1.60
*df*	507	507	507
Religiosity			
Secular (*n* = 205)	3.76 (1.16)^(3)^^*^	9.85 (9.28)	2.36 (0.82)
Traditional (*n* = 137)	3.92 (1.24)^(3)^^*^	8.58 (8.67)	2.47 (0.82)
Religious (*n* = 86)	3.33 (1.21)^(1)^^*^^,^^(2)^^*^	7.67 (9.84)	2.69 (0.93)
*F*	6.64^**^	1.91	4.77^*^
*df*	2, 425	2, 425	2, 425
Educational level			
Up to secondary level (*n* = 291)	3.65 (1.15)	8.93 (9.59)	2.47 (0.84)
Academic level (*n* = 217)	3.87 (1.87)	9.91 (9.22)	2.39 (0.85)
*t*	−2.02^*^	−1.15	1.08
*df*	506	506	506

1 *Secular*.

2 *Traditional*.

3 *Religious*.

* *p < 0.05*,

** *p < 0.01*,

*** *p < 0.001*.

### SEM for Prediction of Individual Function

**Figure 3:**
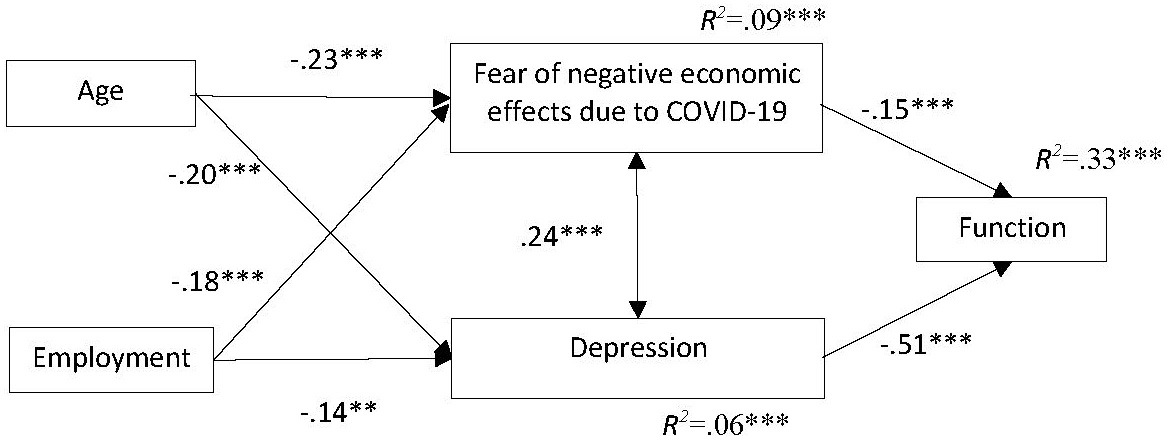
SEM for prediction of individual function during coronavirus quarantine by age, employment, economic fear, and depression. *p < 0.05, **p < 0.01, ***p < 0.001.

We used structural equation modeling (SEM) to test if the hypothesized model (Figure 1) was concordant with the collected data. The variables' correlations are presented in Table 4, and it appears that the correlations among variables were consistent with the expectations. Although the descriptive data indicated that family status is associated with depression and function, the authors did not add it to the model since there was a relatively strong, significant and positive correlation between age and family status [*r*_Spearman_ = 0.31, *p* < 0.001]. The results showed a relatively good fit between our model and the data [$\chi_{\left(3\right)}^{2}$ = 6.1, *p* = 0.11, χ^2^/*df* = 2.03, *CFI* = 0.998, *NFI* = 0.977, *TLI* = 0.939, *RMSEA* = 0.045]. However, within the model, the correlations between fear of COVID-19 and employment [*r* = 0.11, *p* = 0.12] and fear of COVID-19 and function [*r* = 0.03, *p* = 0.42] were statically non-significant. Consequently, the study examined new models in which, instead of the mean scores in fear of COVID-19, the analysis addressed each fear separately. The models that included items on fear of contracting or dying from coronavirus yelled the same results as the original model (Supplementary Table 1). Finally, the authors examined the model that included the fear of negative economic effects following COVID-19 (a.k.a. economic fear). This model was not only a better fit to the data [$\chi_{\left(3\right)}^{2}$ = 4.32, *p* = 0.23, χ^2^/*df* = 1.44, *CFI* = 0.996, *NFI* = 0.986, *TLI* = 0.978, *RMSEA* = 0.029], but also indicated significant correlations between all variables. Figure 3 shows that there is a significant negative correlation between age and depression, and economic fear. The increase in age is associated with a decrease in fears of economic fear and depression. Also, there is a significant negative correlation between employment and economic fear and depression. Respondents who continued working (as usual or from home) were less worried about economic ramifications and reported lower levels of depression. Fear of negative economic effect correlates positively with depression: the higher the level of fear, the higher the level of depression. Finally, economic fear and depression account for 33% of the variance in individual function: the higher the fear and the depression, the lower the individual function level.

**Table 4:** Association between demographic characteristics, function (POAMS-TV) and depression (*N* = 509).

	**1**	**2**	**3**	**4**	**5**	**6**
1. Age	–					
2. Employment	−0.01	–				
3. Fear of COVID-19	−0.14^**^	−0.04	–			
4. Economic fear	−0.21^***^	−0.20^**^	−0.59^***^	–		
5. Depression	0.21^**^	−0.12^**^	−0.30^***^	0.33^***^	–	
6. Function	0.18^**^	0.13^**^	−0.18^***^	−0.29^***^	−0.55^***^	–

** *p < 0.01*,

*** *p < 0.001*.

### Multiple Mediation Effect on the Relationship Between Age and Function

**Figure 4:**
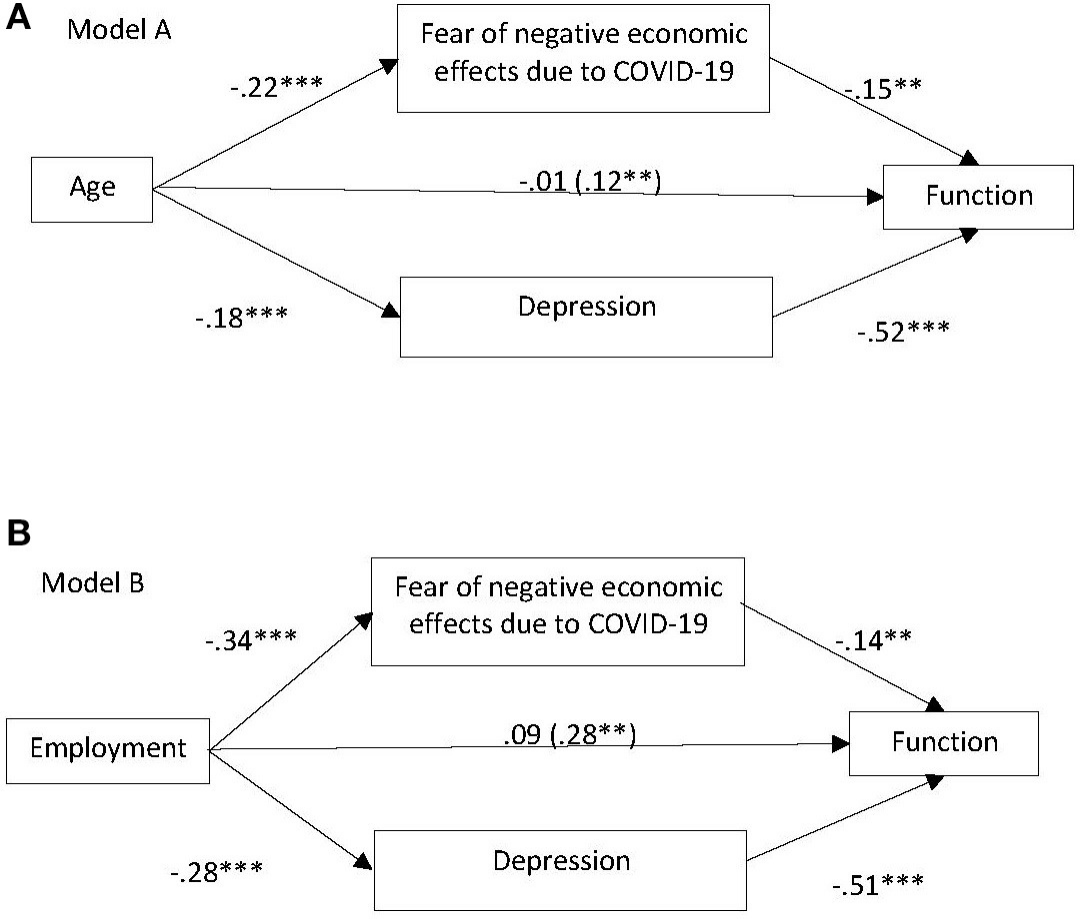
Mediation of economic fear and depression on **(A)** the relationship between age and function; **(B)** the relationship between employment and function. *p < 0.05, **p < 0.01, ***p < 0.001.

Additionally, the study examined multiple mediation effects of economic fear and depression on the relationship between age and function. In step 1 of the mediation model, regressing age on the mediators. The regression of *age* on the mediator, *depression*, was significant (β = −0.18, *p* < 0.001), bootstrap Confidence Interval (CI) range [−0.16, −0.06]. The regression of *age* on the mediator, *economic fear*, was also significant (β = −0.22, *p* < 0.001), bootstrap CI range [−0.03, −0.01]. In step 2, the regression of *age* on the independent variable *function* was significant (β = 0.12, *p* < 0.01), bootstrap CI range [0.002, 011]. Step 3 of the analyses revealed that controlling for both mediators (depression and economic fear), *age* was not a significant predictor of *function* (β = −0.01, *p* > 0.05). The bootstrapped 95% CI for the indirect effect ranged for the mediator *economic fear* [0.001, 0.003], and the mediator *depression* [0.003–0.007], indicating that both indirect effects are significant, since zero did not exist between their CI range (see Model A in Figure 4).

Finally, to investigate which of the two mediators is stronger in the mediation, the authors generated the contrast between the mediators. The bootstrapped 95% CI for the contrast's indirect effect ranged between [0.001, 0.006], indicating a statistical significance. The indirect effect of the mediator depress 2 was 0.005, while the indirect effect of *economic fear* was 0.002, implying that *depression* is stronger than *economic fear* in the mediation process.

### Multiple Mediation Effect on the Relationship Between Employment and Function

In step 1 of the mediation model, regressing *employment* on the mediators. The regression of *employment* on the mediator, *depression*, was significant (β = −0.28, *p* < 0.01), bootstrap Confidence Interval (CI) range [−4.27, −1.011]. The regression of *employment* on the mediator, *economic fear*, was also significant (β = −0.34, *p* < 0.01), bootstrap CI range [−0.82, −0.27]. In step 2, the regression of *employment* on the independent variable *function* was significant (β = 0.12, *p* < 0.01), bootstrap CI range [0.002, 011]. Step 3 of the analyses revealed that controlling for both mediators (depression and economic fear), *employment* was not a significant predictor of *function* (β = 0.09, *p* > 0.05). The bootstrapped 95% CI for the indirect effect ranged for the mediator *economic fear* [0.019, 0.088] and the mediator *depression* [0.054–0.234], indicating that both indirect effects are significant (see Model B in Figure 4).

Finally, to investigate which of the two mediators is stronger in the mediation, the authors generated the contrast between the mediators. The bootstrapped 95% CI for the contrast indirect effect ranged between [0.006, 0.189], indicating a statistical significance. The indirect effect of the mediator *depression* was 0.14, while the indirect effect of *economic fear* was 0.05, implying that *depression* is stronger than *economic fear* in the mediation process.

## Discussion

This study indicates that Israeli citizens reported relatively high levels of depression and fears during coronavirus lockdown and relatively low functioning levels. These findings support the notion that lockdowns and social isolation are associated with adverse psychological reactions (Brooks et al., 2020; Shapiro et al., 2020). Furthermore, the authors constructed a model that included age, employment, fear of COVID-19, and depression. The data mostly supported our hypotheses. This study's main findings indicate that depression and COVID-19 economic fear fully mediate the relationship between age and individual function and the relationship between employment and individual function, with depression being the stronger mediator.

Although older adults are more susceptible to the negative physical effects of the COVID-19 than young adults (Qiu et al., 2020), our results, in line with prior studies, suggest that during the lockdown, the correlation between age and depression and functioning is negative: younger individuals experienced higher levels of depression than older adults. It may be that the risk of viral infection and possible subsequent death are less affecting individual mental and emotional state than the lockdown and social isolation. Older adults, who are more used to staying at home and being alone than young adults (Fogel, 1992; Conejero et al., 2018), maybe less prone to experience depressive symptoms and decreased function than young adults who are at greater risk of psychological distress and loneliness during COVID-19 lockdowns than older adults (Losada-Baltar et al., 2020). Also, older adults may be more psychologically resilient to such unpredictable threats than young adults (Bonanno et al., 2006) or possess superior emotion regulation and coping strategies (Charles, 2010).

Our findings on fears about COVID-19 further support the above suggestion that the fear of illness and death do not affect individuals as much as the lockdowns. Thus, only the economic fear together with depression mediates the relationship between age and function and between employment and function. This pattern may represent the public's understanding that while most of the population is not at risk of dying due to COVID-19 (Cai et al., 2020), and the effects of the illness are relatively temporary (Cai et al., 2020), pandemic's economic effects are much more prominent (Estrada and Arturo, 2020; Jorda et al., 2020).

Our findings regarding the relationship between age and fears of COVID-19 imply that although the economic fallout due to COVID-19 may affect most of the population, its direct effects (e.g., unemployment) are less relevant for older adults who may already have a pension and higher economic security. The salience of the economic loss within the context of viral victimization may also account for the relationship between unemployment, fears, and depression. As it was hypothesized and similarly to prior research (Bijlsma et al., 2017), the unemployed participants reported higher levels of fear and depression and subsequently lower function levels.

### Limitations

The present study is not without limitations. One such limitation is the cross-sectional nature of our data. Therefore, our results indicate an association between the variables, and further research is needed to support the findings on causal pathways between age, employment, fear of COVID-19, depression, and functioning. Secondly, we conducted the survey at the early stages of the COVID-19 pandemic in Israel, and therefore, the findings may represent a result of pandemic-related shock rather than posttraumatic response. Thirdly, the study collected the data through an online panel. A door-to-door sample may differ in the level of depression and functioning. However, the COVID-19 lockdown did not facilitate this method of data collection. Moreover, research indicates that online panels can produce high-quality data (e.g., Blom et al., 2015).

Furthermore, this study is based on self-reports; therefore, the results represent participants' subjective assessment of their function, depression, and fears. Additionally, the findings' external validity may be somewhat limited because the sample did not match the Israeli population regarding ethnicity and academic level. Finally, cultural norms are associated with the tendency to report depression and fears (e.g., Soto et al., 2011), while the structure of social benefits and the scope of government assistance may affect economic uncertainty (Nicola et al., 2020). Therefore, future studies should consider examining our model within other cultural contexts or a cross-cultural framework.

### Conclusions

The study's contributions are both theoretical and practical. It contributes to the body of literature by presenting a model for predicting functioning during pandemic-related lockdowns. According to our model, age and employment are related to economic fears and depression. Together, economic fears and depression predicted the level of individual functioning. These results suggest that there is the age-related victimization paradox during viral pandemics: older adults are more at risk for physical harm but express lower levels of fear and depression. Future studies should examine this paradox. Also, the findings emphasize the significance of employment to the quality of functioning and suggest that the economic implications of COVID-19 affect people more than fear of the virus.

As for practical implications for preventive and therapeutic work during lockdowns, the authors suggest that practitioners who are responsible for public mental health should design programs for such groups at risk as young and unemployed adults. Some of the programs can be based on social support that is a significant protective factor (Saltzman et al., 2020) in situations of isolation or trauma. Moreover, governments should invest in a search for solutions to the mental and economic aspects of viral victimization.

## Data Availability Statement

The raw data supporting the conclusions of this article will be made available by the authors, without undue reservation.

## Ethics Statement

The studies involving human participants were reviewed and approved by Ariel University Ethic Committee. The patients/participants provided their written informed consent to participate in this study.

## Author Contributions

The contribution of IL and KC-L to this study's conception, design, questionnaire development and allocation of funds, and writing and reviewing of the manuscript was equal. IL led the organizing and analyzing of the database. All authors contributed to the article and approved the submitted version.

## Conflict of Interest

The authors declare that the research was conducted in the absence of any commercial or financial relationships that could be construed as a potential conflict of interest.
