# Supplementary material for: Predicting Individual Function During COVID-19 Lockdown: Depression, Fear of COVID-19, Age, and Employment
Source: Front Psychol. 2021 Jul 1;12:682122. doi: 10.3389/fpsyg.2021.682122 (PMC8280345; doi:10.3389/fpsyg.2021.682122)
Supplement: Supplementary file 1 [file Table_1.pdf]

Supplement Materials

Table S1

Standard Regression Weights and Indices for the Different Models

Results	Models				
	1	2	3	4	5
χ^2	6.096	6.252	6.262	6.248	6.238
df	3	3	3	3	3
p	.11	.10	.10	.10	.10
χ^2/df	2.032	2.08	2.087	2.083	2.079
CFI	.988	.985	.987	.985	.987
NFI	.977	.974	.976	.974	.976
TLI	.939	.927	.935	.927	.935
RMSEA	.045	.046	.046	.046	.046
β for the correlation between:					
Age and Fear of COVID-19	-.17***	-.05	-.21***	.05	-.20***
Age and Depression	-.21***	-.21***	-.21***	-.21***	-.21***
Employment and Fear of COVID-19	-.07	-.02	-.03	-.04	.01
Employment and Depression	-.14**	-.14**	-.14**	-.14**	-.14**
Fear of COVID-19 and Function	-.03	.02	.003	-.01	.01
Depression and Function	-.55***	-.56***	-.56***	-.55***	-.56***

*p<.05; **p<.01; ***p<.001

Model 1 – Mean score on fear of COVID-19

Model 2- Fear about contracting COVID-19

Model 3- Fear about a family member contracting COVID-19

Model 4- Fear about dying from COVID-19

Model 5- Fear about a family member dying from COVID-19
